# One-step real-time RT-PCR assays for serotyping dengue virus in clinical samples

**DOI:** 10.1186/s12879-015-1226-z

**Published:** 2015-11-02

**Authors:** Erik Alm, Gunnel Lindegren, Kerstin Ingrid Falk, Nina Lagerqvist

**Affiliations:** Department of Microbiology, The Public Health Agency of Sweden, SE-171 82, Solna, Sweden; Department of Microbiology, Tumor and Cell Biology, Karolinska Institutet, SE-171 77, Stockholm, Sweden

**Keywords:** Dengue virus, Serotype, RT-PCR, Laboratory diagnosis, Serotyping

## Abstract

**Background:**

Dengue is one of the leading causes of morbidity in tropical and subtropical regions and infection with any of the four dengue virus serotypes (DENV1-4) result in a wide range of clinical manifestations. Given the geographic expansion of DENV1-4, assays for serotyping are needed to be able to perform surveillance and epidemiological studies. In this study, we describe the design and validation of one-step real-time serotype-specific DENV RT-PCR assays.

**Methods:**

The DENV1, DENV2, DENV3, and DENV4 RT-PCR assays were designed using all available whole genome DENV sequences in the NCBI nucleotide collection. Because of the high mutation rates of RNA viruses, the assays were performed in singleplex format to enable quick modifications to the primer and probe sequences when new genetic variants emerge. The analytical performance of the RT-PCR assays were evaluated using in vitro transcribed RNA and their specificity was determined by testing 24 DENV isolates, external DENV control panels and RNA preparation of non-DENV flaviviruses and non-dengue clinical samples. Additionally, the clinical performance of the serotype-specific DENV RT-PCR were compared to that of the CDC DENV-1-4 RT-PCR using 85 clinical samples collected from patients presenting with acute dengue.

**Results:**

The RT-PCR assays were found to be specific for their respective serotype and did not cross-react with other flaviviruses or human mRNA. All assays had a linear dynamic range of 10^2^ to 10^6^ copies/reaction with detection limits between 12 and 44 copies/reaction. When testing sera from 85 confirmed acute dengue cases, the serotype-specific DENV RT-PCR assays had 100 % positive agreement with the FDA-approved CDC DENV-1-4 RT-PCR assay performed in a singleplex format. Additionally 15 samples that tested negative in the CDC DENV-1-4- RT-PCR assay were found positive using the serotype-specific DENV RT-PCR assays.

**Conclusions:**

Our results suggest that these RT-PCR assays are useful alternatives to existing methods for serotyping DENV in clinical sera.

**Electronic supplementary material:**

The online version of this article (doi:10.1186/s12879-015-1226-z) contains supplementary material, which is available to authorized users.

## Background

Dengue is the most widespread mosquito-borne viral disease with an estimated 390 million infections yearly [1, 2]. In the last decades, the global distribution of dengue virus (DENV) has expanded to include more geographic areas and all four serotypes (DENV1-4) are now present in Asia, Africa, and the Americas [3]. Not only is dengue a public health concern in endemic regions, dengue has also been increasingly reported in returning travelers [4, 5]. Infection with any of the DENV serotypes is commonly asymptomatic or presented as a feverish illness accompanied by severe headache, muscle and joint pains, and vomiting [6, 7]. Approximately 500 000 cases annually require hospitalization [3]. Severe dengue is characterized by abdominal pain, bleeding, fatigue, and persistent vomiting, and can lead to deadly complications [8, 9].

Acute dengue can be diagnosed by detecting the DENV genome. A number of real-time RT-PCR assays have previously been developed [10–14], including a universal DENV real-time RT-PCR designed and validated at our laboratory [15]. Methods detecting DENV genomes are recommended by the World Health Organization to be used for laboratory confirmation of dengue during the first five to six days after symptomatic onset [16] and PCR-based techniques are at present the only methods for determining the infecting serotype during acute disease.

Here we present the development and clinical validation of four DENV serotype-specific real-time RT-PCR assays useful in situations requiring serotyping. Like many RNA viruses, DENV displays considerable genetic diversity [17]. Consequently, these serotype-specific RT-PCR assays were set up in singleplex format, allowing easy modification of the individual assays when new genetic variants emerge. The DENV1, DENV2, DENV3, and DENV4 real-time RT-PCR assays were designed using all DENV1 (*n* = 1532), DENV2 (*n* = 1117), DENV3 (*n* = 832), and DENV4 (*n* = 145) whole genome sequences available in the NCBI nucleotide collection at the design stage. The performance and specificity of the assays were evaluated by analyzing in vitro transcribed RNA, DENV isolates, and external control panels. Using 85 serum samples obtained from travelers returning from the tropics presenting with acute dengue, the DENV1, DENV2, DENV3 and DENV4 RT-PCR assays were compared to the CDC DENV-1-4 RT-PCR assay [10]. The theoretical and clinical validation of these DENV serotype-specific RT-PCR assays suggest that they are good alternatives to existing methods for serotyping DENV in clinical samples.

## Methods

### Primer and probe design

To be able to find serotype-specific conserved regions, all whole genome sequences of DENV1-4 available at the design stage (2014–10–31) were downloaded from NCBI and used for assay design. Multiple sequence alignments containing the genomic sequences of the individual serotypes and alignments containing all DENV genomic sequences were created using CLC Genomics Workbench 7.5 (www.clcbio.com). Primers and probes were constructed using in-house software. Melting temperatures (Tm) were verified using Primer Express® v3.0 (Applied Biosystems®). Theoretical specificity of the systems was investigated using BLAST against the NCBI nucleotide database with very loose match criteria (word-size = 7, E-cutoff = 1000, match/mismatch cost +1/-1, gap cost 5/2).

### RNA extraction

Viral RNA was extracted from 140 μL supernatant of infected cells or from patient sera using QIAamp® viral RNA extraction kit (Qiagen), following the protocol from the manufacturer. RNA was eluted in 60 μL elution buffer and stored at –80 °C pending analysis. Infectious cell supernatant was subjected to Trizol LS®/chloroform treatment following the manufacturer’s instruction (Invitrogen™) prior to RNA extraction. To confirm the integrity of the extraction reagents and the successful recovery of RNA from clinical samples, the presence of human beta-actin mRNA was analyzed using a commercial TaqMan® probe-based RT-PCR assay (Applied Biosystems®).

### One-step real-time RT-PCR assays

The DENV1, DENV2, DENV3, and DENV4 RT-PCR assays were carried out in 25 μL reaction mixtures containing 5 μL template RNA, TaqMan® Fast Virus 1-step mastermix (Applied Biosystems®), UltraPure™ DNase/RNase-Free Distilled Water (Invitrogen™), 0.9 μM of each primer, and 0.2 μM probe (Table 1). The MGB-probes were labeled with FAM reporter dye and a non-fluorescent quencher. Primers and probes were purchased from Life Technologies. Amplification and detection were performed in a StepOne Plus real-time PCR system (Applied Biosystems®). Thermocycling parameters were as follows: reverse transcription at 50 °C for 5 min, inactivation at 95 °C for 20 s, followed by 45 cycles of fluorescence detection at 95 °C for 3 s, and annealing at 60 °C for 30 s. The baseline and threshold were set using the auto-baseline and threshold feature in StepOne Software v2.2.2 (Applied Biosystems®). Samples were considered positive if target amplification was recorded within 40 cycles.

**Table 1:** Primer and probe sequences

Assay	Primer/probe	Amplicon size (nt)	Target region	Sequences (5’-3’)	Position	Tm
DENV1	DENV1_F	71	NS5	CAATGGATGACAACAGAAGAYATG	9974–9997	56.6
	DENV1_R			TCCATCCATGGGTTTTCCTCTAT	10022–10044	59.5
	DENV1_P			TCAGTGTGGAATAGGGTTT	10001–10019	70.0
DENV2	DENV2_F	199	E protein	GCAGAAACACAACATGGAACRATAGT	1873–1898	56.6
	DENV2_R			TGATGTAGCTGTCTCCRAATGG	2050–2071	59.8
	DENV2_P			TCAACATAGAAGCAGAACC	2030–2048	68.0
DENV3	DENV3_F	167	NS1	ATGGAATGTGTGGGAGGTGG	2860–2879	59.1
	DENV3_R			GGCTTTCTATCCARTAGCCCATG	3004–3026	59.8
	DENV3_P			TATGGCTGAAACTCCGAG	2913–2930	68.0
DENV4	DENV4_F	114	5’ UTR/	GCAGATCTCTGGAAAAATGAACCA	86–109	60.4
	DENV4_R		capsid	GAGAATCTCTTCACCAACCCYTG	177–199	59.8
	DENV4_P		protein	TCAATATGCTGAAACGC	136–152	68.0

Degenerated nucleotides (nt): R: A/G, Y: G/A/C. The MGB-modified probes were labelled with FAM and a non-fluorescence quencher (NFQ)

Genome positions are given according to the NCBI reference sequence for DENV1 [GenBank:NC_001477], DENV2 [GenBank:NC_001474], DENV3 [GenBank:NC_001475], and DENV4 [GenBank:NC_002640]

The mean melting temperatures (Tm) are shown for primers with degenerated nucleotides

The CDC DENV-1-4 Real-Time RT-PCR Assay [10] was performed in singleplex reactions following the manufacturer’s instructions (Centers for Disease Control and Prevention) in 25 μL volumes using the SuperScript® III Platinum® One-Step qRT-PCR Kit (Invitrogen™). Amplification and detection were performed in a 7500 Fast DX Real-time PCR instrument (Applied Biosystems®). The data analysis were performed as described in the manufacturer’s guidelines. In short, the threshold was adjusted to fall within the PCR exponential phase in the linear view. The manufacturer’s instructions specifies that a specimen is considered positive for either DENV1, 2, 3, or 4 if the amplification curve crosses the threshold line within 37 cycles (Cq < 37).

### Amplification efficiency, linear dynamic range, and limit of detection

The efficiency of amplification and the dynamic range were determined by testing triplicates of 10-fold serial dilutions of in vitro transcribed RNA (BioSynthesis Inc.). The RNA transcripts were 158, 235, 189, and 133 nt in length and were based on the sequences of DENV1 [GenBank:GU131828], DENV2 [GenBank:EU482743], DENV3 [GenBank:AY679147], and DENV4 [GenBank:GQ199881], respectively. The RNA was diluted in RNase/DNase-free water (Life Technologies) and stored at –80°C. The RNA concentration was determined by the manufacturer and verified using a Nano Drop ND-1000 spectrophotometer (Thermo Scientific).

The limit of detection (LoD) was determined by testing two-fold serially diluted in vitro transcribed RNA close to the detection limit of the assay. The transcript RNA was tested in eight replicates in three separate experiments and LoD was defined as the last dilution in which a fluorescence signal could be detected in all 24 replicates. Dilutions and other pipetting procedures were performed using a robotic QIAgility™ workstation (Qiagen).

### Specificity study

DENV used in this study was DENV1 (8356/10, Hawaii, and West Pac.), DENV2 (4397/11 and NewGuinea C), DENV3 (3140/09 and H-87), DENV4 (3274/09 and H-241) and 15 partly sequenced DENV isolates: DENV1 (*n* = 8), DENV2 (*n* = 3), and DENV3 (*n* = 4). The following 12 non-DENV agents were selected for the specificity validation: Andes virus (ANDV), chikungunya virus (CHIKV), hantaan virus (HTNV), Lassa virus (LASV), Rift Valley fever virus (RVFV), Seoul virus (SEOV), Dobrava-Belgrade virus (DOBV), tick-borne encephalitis virus (TBEV), Usutu virus (USUV), West Nile virus (WNV), yellow fever virus (YFV), and Zika virus (ZIKV). Hantaviruses were propagated in Vero E6 cells (ATCC: 1586) in Minimal Essential Medium supplemented with 5 % fetal bovine serum, 1 mM HEPES, and 100 U/mL penicillin, and 100 μg/mL streptomycin. CHIKV, DENV, LASV, RVFV, TBEV, USUV, WNV, YFV, and ZIKV were propagated in Vero cells (ATCC: CCL-81) in Medium 199 supplemented with 5 % fetal bovine serum, 1 mM HEPES, and 100 U/mL penicillin, and 100 μg/mL streptomycin. All reagents were from Gibco, Life Technologies. Cells were maintained at 37 °C in a humidified atmosphere containing 5 % CO2. All handling of infectious material was performed in bio-safety level (BSL) 3, and for LASV, BSL 4 containment laboratories. External DENV control panels were obtained from Quality Control for Molecular Diagnostics (QCMD) in the years of 2011, 2012, 2013, and 2014.

### Clinical samples

Serum samples (*n* = 85) collected during 2014–2015 from Swedish residents returning from travel with confirmed dengue were obtained from the biorepository of the Public Health Agency of Sweden as stipulated in the regulations for use of such material in diagnostic development and quality assessment. The Public Health Agency of Sweden performs all dengue diagnostics in Sweden and the biobank deposited samples used in this study had previously been confirmed DENV positive by using a universal one-step real-time DENV RT-PCR [15] and the SD Bioline Dengue NS1 Ag Rapid Test (Standard Diagnostics, Inc.). Additionally, all patients had been confirmed as having dengue using Panbio® Dengue IgM capture ELISA (Alere™) and in a few cases an in-house IFA [18]. Serum samples (*n* = 40) collected during 2014 from non-dengue cases were used for testing possible unspecific reactions of the DENV1, DENV2, DENV3, and DENV4 real-time RT-PCR assays with human RNA. No informed consent or ethical permit was required since The Swedish Ethical Review Act (2003:16), Ethical Review of Research Involving Humans (http://www.epn.se/media/1205/the_ethical_review_act.pdf) is not applicable for diagnostic development and quality assessment.

## Results and discussion

The primers and probes were designed using 1532 DENV1, 1117 DENV2, 832 DENV3, and 145 DENV4 complete genome sequences. To minimize the possibility of unspecific detection of non-targeted serotypes, the individual RT-PCR assays were designed to have a minimum number of mismatches to the targeted DENV serotype (Additional file 1) while a maximum number of mismatches in the 3’ region of the primers and evenly distributed mismatches in the probe in respect to the sequences of non-targeted serotypes (Additional file 2). The sequences of the primers and probes can be found in Table 1. BLAST against all non-DENV sequences in the NCBI Nucleotide Collection indicated no risk of cross-reaction with other species.

**Fig. 1:**
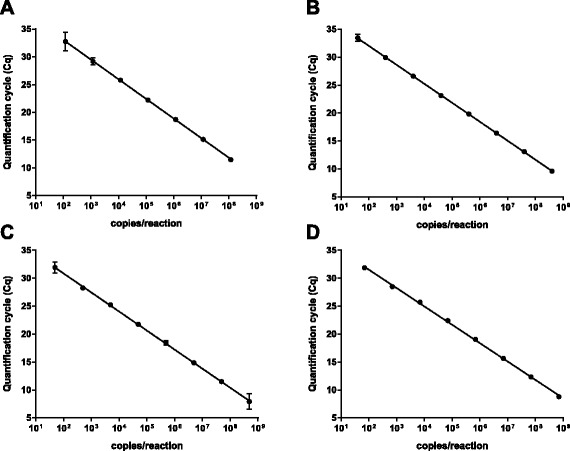
Linear dynamic range. The linear dynamic ranges of the RT-PCR assays were determined by testing triplicates of 10-fold serially diluted in vitro transcribed RNA. Panel (**a**) represents the DENV1 assay, and (**b**), (**c**), and (**d**) the DENV2, DENV3, and DENV4 RT-PCR assays, respectively. Each dot represents the mean Cq-value from three replicates, the error bars indicate 95 % confidence intervals, and the lines represents the best fitting lin-log regression models

To determine the amplification efficiency and the linear dynamic range, in vitro transcribed RNA was 10-fold serially diluted and tested in triplicates in the individual assays. All assays had a linear dynamic range from 10^2^ to 10^8^ copies/reaction (Fig. 1). Over this interval the amplification efficiency was 96 % (R^2^ = 0.999, y-intercept = 37.4), 97 % (R^2^ = 1, y-intercept = 38.8), 97 % (R^2^ = 0.999, y-intercept = 37.7), and 102 % (R^2^ = 0.999, y-intercept = 38.1) for the DENV1, DENV2, DENV3, and DENV4 RT-PCR assay, respectively (Fig. 1).

**Fig. 2:**
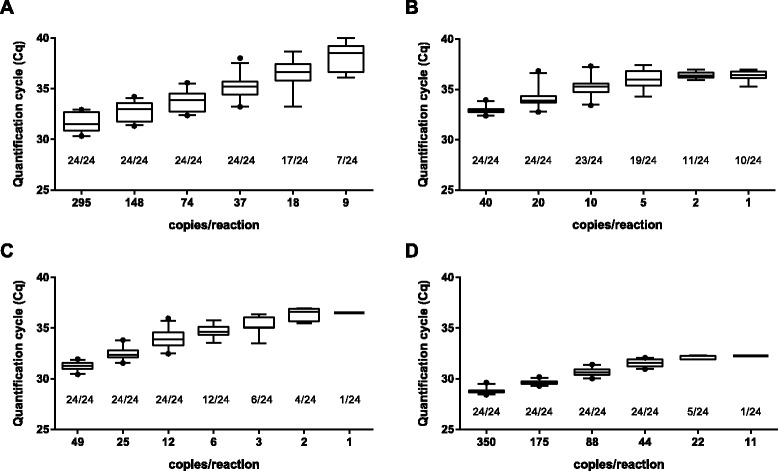
Limit of detection. Limit of detection was determined by assaying eight replicates of twofold serially diluted RNA transcripts in three separate experiments. The number of positives per total number of replicates tested is shown for (**a**) the DENV1 RT-PCR assay, (**b**) the DENV2 RT-PCR assay, (**c**) the DENV3 RT-PCR assay, and (**d**) the DENV4 RT-PCR assay. Horizontal lines indicate mean values, boxes denote the 25th to 75th percentiles, whiskers the 5–95 % percentiles, and dots represent outliers

LoD was determined by testing two-fold serial dilutions of in vitro transcribed RNA in 24 replicates (Fig. 2). The detection limits for the one-step real-time DENV1, DENV2, DENV3, and DENV4 RT-PCR assays were 37 copies/reaction (Fig. 2a), 20 copies/reaction (Fig. 2b), 12 copies/reaction (Fig. 2c), and 44 copies/reaction (Fig. 2d), respectively.

The assays were evaluated for their specificity by testing 24 DENV isolates and four external QCMD control panels, each including 10 samples containing different concentrations of DENV and two negative controls (Table 2). The four serotype-specific RT-PCR assays correctly classified all samples with no observed cross-reactions (Table 2). To confirm that the primers and probes of the DENV1, DENV2, DENV3, and DENV4 RT-PCR assays did not react with human RNA, RNA preparations of 40 serum samples obtained from patient with non-dengue diagnosis were analyzed. All of these samples contained mRNA of human beta-actin, indicating adequate RNA extraction. No signal was observed in the DENV1, DENV2, DENV3, and DENV4 RT-PCR assays.

**Table 2:** Specificity of the DENV1, DENV2, DENV3, and DENV4 real-time RT-PCR assays

Sample	No. of positive samples/no. tested			
	DENV1 RT-PCR	DENV2 RT-PCR	DENV3 RT-PCR	DENV4 RT-PCR
DENV1 isolates	11/11	0/11	0/11	0/11
DENV2 isolates	0/5	5/5	0/5	0/5
DENV3 isolates	0/6	0/6	6/6	0/6
DENV4 isolates	0/2	0/2	0/2	2/2
QCMD panel, DENV1	17/17	0/17	0/17	0/17
QCMD panel, DENV2	0/11	11/11	0/11	0/11
QCMD panel, DENV3	0/8	0/8	8/8	0/8
QCMD panel, DENV4	0/4	0/4	0/4	4/4
QCMD negative controls	0/8	0/8	0/8	0/8

RNA preparations from DENV1, DENV2, DENV3, and DENV4 isolates obtained Cq-values between 16 and 25 when analyzed using their respective serotype-specific RT-PCR assay

The concentrations of DENV1, DENV2, and DENV3 included in the QCMD panels were 10^3^ to 10^6^, 10^2^ to 10^5^, and 10^3^ to 10^5^ copies/mL, respectively. The concentration of DENV4 was 10^5^ copies/mL in all four QCMD panel samples

The differential diagnosis of dengue can be challenging due to the wide range of clinical manifestations [6, 9]. For this reason, the specificity of the RT-PCR assays were further evaluated by testing RNA extracted from high-titred solutions of other flaviviruses and viruses important for dengue differential diagnosis. No unspecific reactions were observed for RNA extracted from preparations of other flaviviruses (TBEV, USUV, WNV, YFV, and ZIKV) or non-flaviviruses (ANDV, CHIKV, DOBV, HTNV, RVFV, LASV, and SEOV).

The serotype-specific DENV RT-PCR assays were evaluated for their clinical performance by retrospectively testing 85 serum samples obtained from patients presenting with acute dengue (Table 3). These 85 serum samples were also tested using the FDA-approved CDC DENV-1-4 RT-PCR (reference method) performed in singleplex format [10] (Table 3). The DENV1, DENV2, DENV3, and DENV4 RT-PCR assay (test methods) results were all in 100 % positive agreement [19] to the CDC DENV-1-4 RT-PCR results , i.e. all samples that were positive in the reference method were also positive in the test methods (Table 3 and Additional file 3). An additional 15 samples not detected by the CDC DENV RT-PCR assay were classified as DENV1 (*n* = 6), DENV2 (*n* = 6), DENV3 (*n* = 2), and DENV4 (*n* = 1) using the DENV1, DENV2, DENV3, and DENV RT-PCR assays, respectively (Table 3 and Additional file 3). Four of these 15 samples were collected between days 4 and 5 after symptomatic onset, the remaining 11 samples were collected days 6–9. The DENV1 and DENV2 assays were found to be more sensitive than the CDC DENV1 and DENV2 assays (Chi-square test, *p* = 0.009 and *p* = 0.003, respectively). The DENV3 and DENV4 assays were not found to be significantly more sensitive than the CDC DENV3 and DENV4 assays (Chi-square test, *p* = 0.11 and *p* = 0.28, respectively). The CDC DENV-1-4 RT-PCR assay has previously been shown to be less sensitive than an in-house developed RT-PCR assay especially for serum samples collected more than 5 days after symptomatic onset [20].

**Table 3:** Comparable analysis between the serotype-specific RT-PCR assays and the CDC DENV1-4 RT-PCR

		CDC DENV1 RT-PCR	
		negative	Positive
DENV1 RT-PCR	negative	36	0
	positive	6	43
		CDC DENV2 RT-PCR	
		negative	positive
DENV2 RT-PCR	negative	67	0
	positive	6	12
		CDC DENV3 RT-PCR	
		negative	positive
DENV3 RT-PCR	negative	76	0
	positive	2	7
		CDC DENV4 RT-PCR	
		negative	positive
DENV4 RT-PCR	negative	78	0
	positive	1	6

A sample was classified as positive in the CDC DENV-1-4 real-time RT-PCR assays if the amplification curve crossed the threshold line within 37 cycles

Five of the six DENV1 RT-PCR positive samples, two of the six DENV2 RT-PCR positive samples, both the DENV3 RT-PCR positive samples, and the DENV4 RT-PCR positive sample that tested negative in respective CDC DENV-1-4 RT-PCR singleplex assay crossed the threshold line between 37 and 40 cycles in the CDC assays

## Conclusions

The four DENV serotype-specific RT-PCR assays presented here were able to successfully serotype dengue in clinical samples. RNA preparations of 40 serum samples obtained from patient with non-dengue diagnosis did not show unspecific reactions, however, it should be noted that the assay have only been evaluated using selected samples i.e. samples already confirmed as DENV positive. No unspecific reactions were observed when high-titred preparations of non-targeted DENV serotypes were analyzed, this may be useful especially considering that co-circulations of several serotypes as well as co-infections with two serotypes have been reported [21–23]. The DENV1 and DENV2 RT-PCR assays were found to be more sensitive than the corresponding FDA-approved CDC DENV assays and the DENV3 and DENV4 were found to be at least as sensitive as the corresponding CDC DENV assays when serum samples from 85 acute dengue cases were analyzed. This indicates that the DENV1, DENV2, DENV3 and DENV4 assays are useful alternatives to other methods for serotyping DENV.

## Additional files

Additional file 1:
**Overview of the primers and probes.** Vertical bars and percentages show the fraction of sequences with nucleotides deviating from the consensus of (A) DENV1, (B) DENV2, (C) DENV3, and (D) DENV4 serotypes. Values below 1 % are not shown. Numbers indicate genomic positions. (PDF 82 kb) Additional file 2:
**Overview of the theoretical specificity towards non-targeted serotypes.** The sequences of the primers and probe in the (A) DENV1, (B) DENV2, (C) DENV3, and (D) DENV4 RT-PCR plotted against their respective non-targeted serotypes. Numbers above the primers and probe indicate genomic positions. (PDF 87 kb) Additional file 3:
**Cq-value comparison between the test methods and reference method.** The table shows the Cq-values obtained for the 85 dengue positive samples tested in the different serotype-specific RT-PCR assays. Information on days after symptomatic onset were included for samples for which this data was available. (PDF 253 kb)

## Abbreviations

ANDV: Andes virus
BLAST: Basic local alignment search tool
BSL: Biosafety level
CDC: Center of Disease Control
CHIKV: Chikungunya virus
Cq: Quantification cycle
DENV: Dengue virus
DENV1-4: Dengue virus serotype 1-4
DOBV: Dobrava-Belgrade virus
FAM: 6-carboxyfluorescein
FDA: Food and Drug Administration
HTNV: Hantaan virus
LASV: Lassa virus
LoD: Limit of detection
MGB: Minor groove binder
NCBI: National Center for Biotechnology Information
NFQ: Non-fluorescence quencher
nt: Nucleotide
QCMD: Quality Control for Molecular Diagnostics
RT-PCR: Reverse transcription polymerase chain reaction
RVFV: Rift Valley fever virus
SEOV: Seoul virus
TBEV: Tick-borne encephalitis virus
Tm: Melting temperature
USUV: Usutu virus
WNV: West Nile virus
ZIKV: Zika virus
