# Supplementary figures and images for: One-step real-time RT-PCR assays for serotyping dengue virus in clinical samples
Source: BMC Infect Dis. 2015 Nov 2;15:493. doi: 10.1186/s12879-015-1226-z (PMC4630907; doi:10.1186/s12879-015-1226-z)

**A**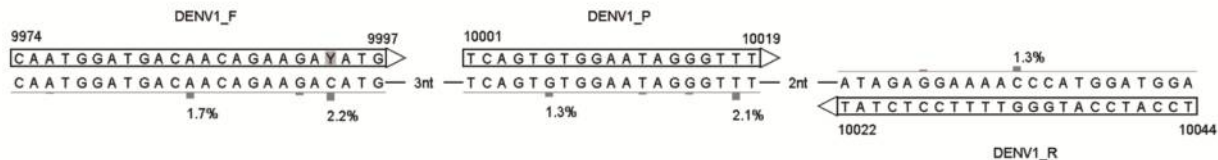**B**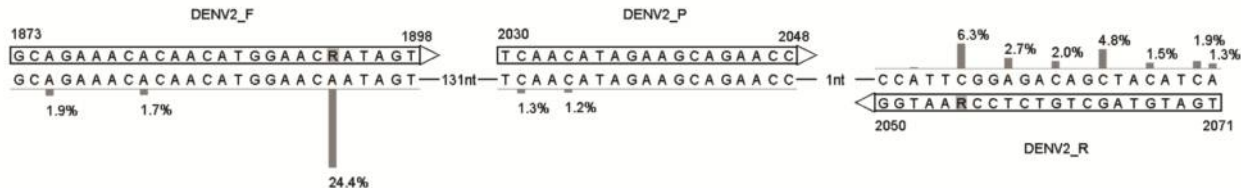**C**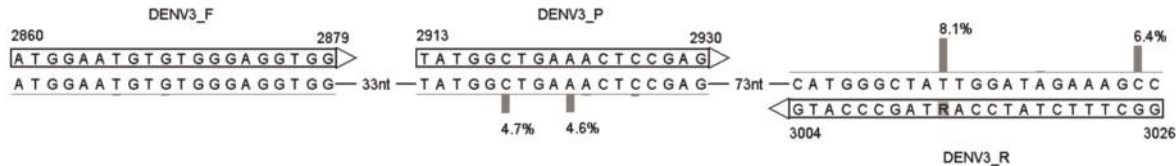**D**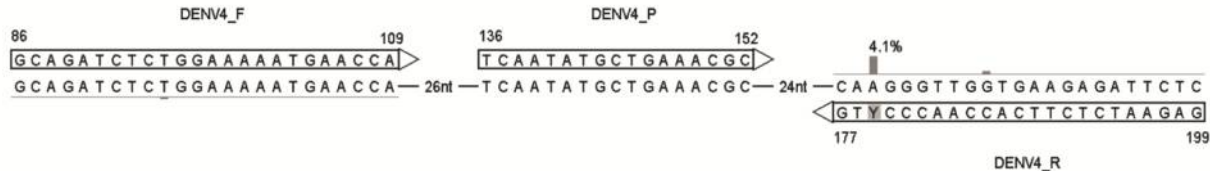

Supplement: Additional file 1: — Overview of the primers and probes. Vertical bars and percentages show the fraction of sequences with nucleotides deviating from the consensus of (A) DENV1, (B) DENV2, (C) DENV3, and (D) DENV4 serotypes. Values below 1 % are not shown. Numbers indicate genomic positions. (PDF 82 kb) [file 12879_2015_1226_MOESM1_ESM.pdf]

**A**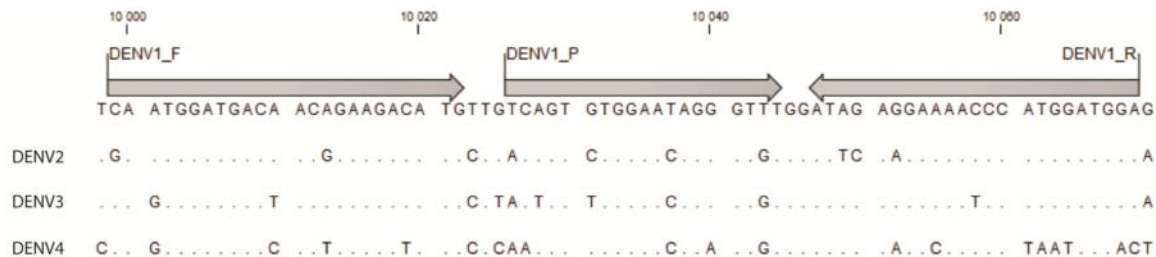**B**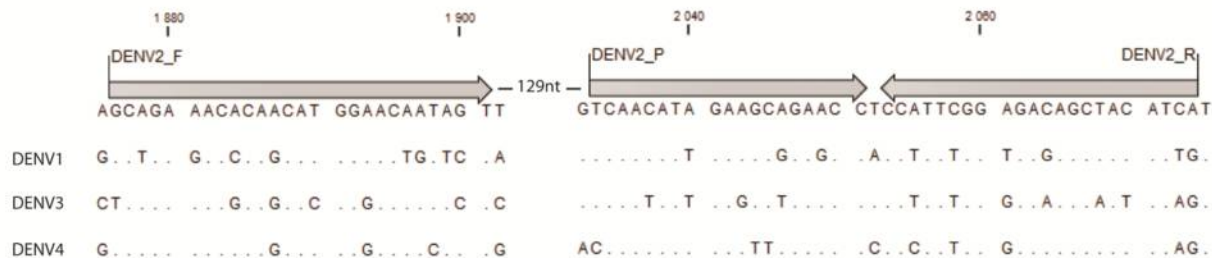**C**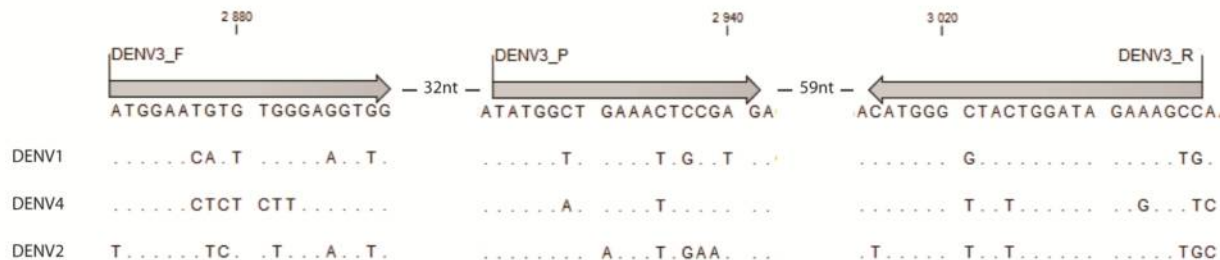**D**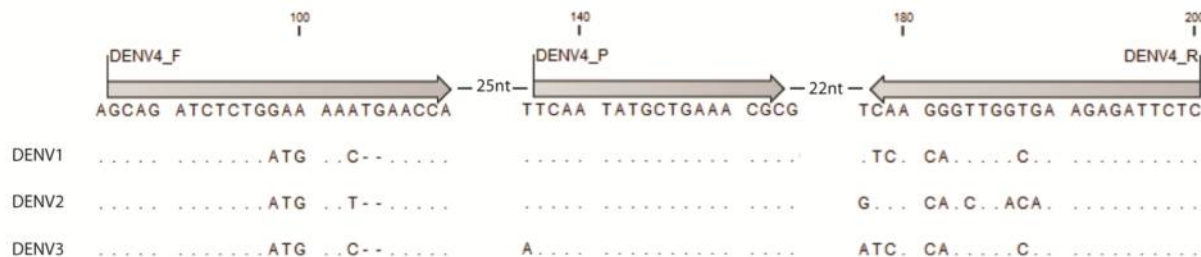

Supplement: Additional file 2: — Overview of the theoretical specificity towards non-targeted serotypes. The sequences of the primers and probe in the (A) DENV1, (B) DENV2, (C) DENV3, and (D) DENV4 RT-PCR plotted against their respective non-targeted serotypes. Numbers above the primers and probe indicate genomic positions. (PDF 87 kb) [file 12879_2015_1226_MOESM2_ESM.pdf]
