# Supplementary material for: One-step real-time RT-PCR assays for serotyping dengue virus in clinical samples
Source: BMC Infect Dis. 2015 Nov 2;15:493. doi: 10.1186/s12879-015-1226-z (PMC4630907; doi:10.1186/s12879-015-1226-z)
Supplement: Additional file 3: — Cq-value comparison between the test methods and reference method. The table shows the Cq-values obtained for the 85 dengue positive samples tested in the different serotype-specific RT-PCR assays. Information on days after symptomatic onset were included for samples for which this data was available. (PDF 253 kb) [file 12879_2015_1226_MOESM3_ESM.pdf]

Additional file 3. Cq-value comparison between the test methods and reference method

Sample nr.	Days after symptom onset*	DENV1	CDC DENV-1	DENV2	CDC DENV-2	DENV3	CDC DENV-3	DENV4	CDC DENV-4
		Test method	Reference method	Test method	Reference method	Test method	Reference method	Test method	Reference method
**1**	4	nd	nd	nd	nd	nd	nd	26.8	27.8
**2**	6	30.2	32.6	nd	nd	nd	nd	nd	nd
**3**	5	30.0	31.6	nd	nd	nd	nd	nd	nd
**4**	6	33.9	38.7	nd	nd	nd	nd	nd	nd
**5**	7	nd	nd	37.2	nd	nd	nd	nd	nd
**6**	3	18.9	21.2	nd	nd	nd	nd	nd	nd
**7**	6	nd	nd	34.9	nd	nd	nd	nd	nd
**8**	5	30.1	35.6	nd	nd	nd	nd	nd	nd
**9**	9	nd	nd	nd	nd	nd	nd	nd	nd
**10**	4	21.5	22.0	nd	nd	nd	nd	nd	nd
**11**	5	nd	nd	nd	nd	nd	nd	30.0	30.7
**12**	9	28.5	32.9	nd	nd	nd	nd	nd	nd
**13**	6	30.7	34.1	nd	nd	nd	nd	nd	nd
**14**	5	nd	nd	33.4	38.0	nd	nd	nd	nd
**15**	3	26.9	29.9	nd	nd	nd	nd	nd	nd
**16**	8	nd	nd	nd	nd	nd	nd	nd	nd
**17**	7	32.7	38.6	nd	nd	nd	nd	nd	nd
**18**	8	34.7	37.6	nd	nd	nd	nd	nd	nd
**19**	4	nd	nd	nd	nd	31.7	37.1	nd	nd
**20**	5	30.2	34.5	nd	nd	nd	nd	nd	nd
**21**	4	22.7	24.0	nd	nd	nd	nd	nd	nd
**22**	6	29.7	36.8	nd	nd	nd	nd	nd	nd
**23**	7	nd	nd	nd	nd	36.0	nd	nd	nd
**24**	7	nd	nd	33.3	34.6	nd	nd	nd	nd
**25**	8	34.4	37.1	nd	nd	nd	nd	nd	nd
**26**	7	30.2	33.1	nd	nd	nd	nd	nd	nd
**27**	4	22.9	26.7	nd	nd	nd	nd	nd	nd
**28**	6	29.7	35.0	nd	nd	nd	nd	nd	nd
**29**	4	23.3	26.9	nd	nd	nd	nd	nd	nd
**30**	6	29.0	32.3	nd	nd	nd	nd	nd	nd
**31**	7	nd	nd	nd	nd	31.9	31.9	nd	nd
**32**	4	21.3	21.0	nd	nd	nd	nd	nd	nd
**33**	4	nd	nd	nd	nd	21.0	28.0	nd	nd
**34**	9	26.9	29.6	nd	nd	nd	nd	nd	nd
**35**	4	nd	nd	31.9	32.4	nd	nd	nd	nd
**36**	6	nd	nd	26.6	28.2	nd	nd	nd	nd
**37**	3	nd	nd	nd	nd	33.0	28.4	nd	nd
**38**	5	24.2	30.8	nd	nd	nd	nd	nd	nd
**39**	8	35	36.2	nd	nd	nd	nd	nd	nd
**40**	3	nd	nd	nd	nd	17.5	20.0	nd	nd
**41**	7	28.4	32.3	nd	nd	nd	nd	nd	nd
**42**	1	nd	nd	11.4	17.9	nd	nd	nd	nd
**43**	4	33.9	37.0	nd	nd	nd	nd	nd	nd
**44**	7	38.3	nd	nd	nd	nd	nd	nd	nd
**45**	6	34.4	39.3	nd	nd	nd	nd	nd	nd
**46**	2	16.8	19.5	nd	nd	nd	nd	nd	nd
**47**	3	nd	nd	nd	nd	17.8	16.0	nd	nd
**48**	n/a	31.7	32.2	nd	nd	nd	nd	nd	nd
**49**	8	33.9	34.3	nd	nd	nd	nd	nd	nd
**50**	n/a	29.3	36.6	nd	nd	nd	nd	nd	nd
**51**	3	nd	nd	nd	nd	23.1	24.3	nd	nd
**52**	3	19.5	22.2	nd	nd	nd	nd	nd	nd
**53**	n/a	nd	nd	nd	nd	nd	nd	28.4	30.0
**54**	5	nd	nd	31.9	36.9	nd	nd	nd	nd
**55**	n/a	nd	nd	24.9	28.4	nd	nd	nd	nd

**56**	5	nd	nd	nd	nd	nd	nd	25.9	25.9
**57**	4	nd	nd	37.9	nd	nd	nd	nd	nd
**58**	n/a	29.8	34.0	nd	nd	nd	nd	nd	nd
**59**	5	32.6	36.6	nd	nd	nd	nd	nd	nd
**60**	1	nd	nd	nd	nd	nd	nd	25.0	26.8
**61**	5	nd	nd	nd	nd	nd	nd	30.5	39.1
**62**	n/a	24.7	26.1	nd	nd	nd	nd	nd	nd
**63**	n/a	23.3	26.2	nd	nd	nd	nd	nd	nd
**64**	4	nd	nd	24.8	23.3	nd	nd	nd	nd
**65**	6	28.4	32.7	nd	nd	nd	nd	nd	nd
**66**	4	22.3	25.4	nd	nd	nd	nd	nd	nd
**67**	3	18.9	22.3	nd	nd	nd	nd	nd	nd
**68**	6	nd	nd	nd	nd	nd	nd	28.5	29.8
**69**	1	nd	nd	20.0	30.8	nd	nd	nd	nd
**70**	4	28.7	31.7	nd	nd	nd	nd	nd	nd
**71**	5	31.2	34.2	nd	nd	nd	nd	nd	nd
**72**	3	nd	nd	21.4	25.7	nd	nd	nd	nd
**73**	6	nd	nd	36.1	nd	nd	nd	nd	nd
**74**	n/a	24.0	25.1	nd	nd	nd	nd	nd	nd
**75**	5	33.7	36.9	nd	nd	nd	nd	nd	nd
**76**	n/a	17.2	20.2	nd	nd	nd	nd	nd	nd
**77**	n/a	nd	nd	31.3	36.8	nd	nd	nd	nd
**78**	9	nd	nd	34.8	39.4	nd	nd	nd	nd
**79**	2	nd	nd	nd	nd	15.0	16.1	nd	nd
**80**	n/a	26.9	30.2	nd	nd	nd	nd	nd	nd
**81**	n/a	25.0	25.4	nd	nd	nd	nd	nd	nd
**82**	n/a	nd	nd	15.1	19.6	nd	nd	nd	nd
**83**	5	28.5	29.4	nd	nd	nd	nd	nd	nd
**84**	5	26.7	29.1	nd	nd	nd	nd	nd	nd
**85**	1	nd	nd	14.9	20.1	nd	nd	nd	nd

Grey boxes represent samples that tested positive in the individual assays: for the DENV1, DENV2, DENV3, DENV4 assays samples with a Cq value < 40 and for the CDC DENV-1-4 assays Cq value < 37 (according to the manufacturer's instructions). Orange boxes represent samples that were classified as negative in the CDC DENV-1-4 assays but had Cq values < 40. Red boxes represent samples which tested positive in the DENV1, DENV2, DENV3, or the DENV4 assay but for which no amplification was recorded in the CDC DENV-1-4 assays.

nd = not detected

n/a = not available
